# A prospective evaluation of subgingival irrigation with i-PRF following non-surgical treatment of peri-implantitis

**DOI:** 10.3389/fdmed.2025.1568889

**Published:** 2025-06-12

**Authors:** Laurie Deterville, Jérôme Frédéric Lasserre, Selena Toma

**Affiliations:** Department of Periodontology, Institut de Médecine Dentaire et de Stomatologie, Cliniques Universitaires Saint Luc, Université catholique de Louvain, Brussels, Belgium

**Keywords:** peri—implantitis, PRF (platelet-rich fibrin), nonsurgical, injectable, air abrasive

## Abstract

**Introduction:**

Peri-implantitis, an inflammatory condition around dental implants, is challenging to manage with conventional non-surgical treatments alone. Emerging adjunctive therapies like glycine air-polishing and injectable platelet-rich fibrin (i-PRF) show potential to enhance decontamination.

**Aim:**

To clinically and radiographically evaluate the efficacy of glycine air-polishing and the adjunctive use of injectable platelet rich fibrin (i-PRF) for the non-surgical treatment of slight peri-implantitis.

**Methods:**

For this prospective case series, nine patients (*n* = 14 implants), with at least one implant with a slight peri-implantitis (radiographic bone loss visible and up to 4 mm) were enrolled. All treated implants received the same treatment: non-surgical mechanical debridement with an air abrasive device (PERIOFLOW®) followed by a subgingival irrigation with an injectable platelet rich fibrin (i-PRF). The following clinical parameters were measured: Plaque Index (PI), Bleeding on Probing (BoP), Suppuration on Probing (SoP), Probing Pocket Depth (PPD), Relative Attachment Level (RAL) and Recession (REC). They were assessed at baseline (M0), 3 and 6 months (M3 and M6). To compare bone level (BL), radiographs were taken at M0 and M6 (*p* > 0.05, ANOVA, Bonferroni).

**Results:**

Results indicated that PI significantly decreased over 6 months to a mean value of <0.05. Both BoP (*p* < 0.05) and SoP (*p* < 0.05) were substantially reduced at 3 months, although a slight increase was noted at 6 months. Mean PPD was 3.61 ± 0.25 mm (*p* < 0.05) at M6, and RAL gain was significantly improved at 6 months (7.76 ± 0.34 mm, *p* < 0.05). BL showed a significant grain at 6 months (*p* < 0.05). Most mucosal recession occurred within the first 3 months, with no significant change at 6 months.

**Conclusion:**

The application of i-PRF after a subgingival debridement using glycine air-polishing shows significant improvement of clinical parameters and a bone level stability for at least six months. However, if we consider that no bleeding on probing is needed to control the disease, none of the implants were considered successfully treated. Further randomized clinical trials are needed to evaluate the benefits of i-PRF as an adjuvant to the treatment of peri-implantitis.

## 1 Introduction

For several decades, dental implants have become a popular and reliable method for replacing missing teeth in both fully (1, 2) and partially edentulous patients (3).

Even if dental implants have been demonstrated as an effective and predictable treatment with an estimated survival rate of 93.2% at 10 years (4), biological complications may occur after osseointegration (5)*.* In most cases, they are caused by the accumulation of pathogenic microbes at the implant-mucosal interface organized in a highly structured biofilm which causes a loss of host-microbe homeostasis (6–8).

Peri-implant mucositis has been described as a reversible inflammation strictly limited to the surrounding soft tissues and characterized by erythema, bleeding with or without suppuration on gentle probing and swelling of the mucosa (9).

It is considered to be the precursor of peri-implantitis which is an irreversible inflammatory disease of the peri-implant tissues associated with a progressive loss of the supporting bone. It is characterized by clinical signs of inflammation with an increased pocket depth and radiographic bone loss (7, 10)*.* Therefore, avoiding the development of deep lesions by early treatment of small lesions may be of interest.

According to the literature, the prevalence of mucositis and peri-implantitis can be as high as 63.4% of patients (30.7% of implants) (11) and 18.5% of patients (12.8% of implants) (12), respectively.

Peri-implant diseases can also be initiated and/or maintained by iatrogenic factors (e.g., excess cementation, over-contouring of the restoration, poorly positioned implant,…) (7, 13)*.* Other potential risk factors for the development of these diseases have been identified such as an untreated periodontitis, smoking, diabetes or lack of keratinized mucosa (14).

The control of risk factors and decontamination of the colonized implant surface is the cornerstone of currently proposed treatment approaches. As a result, an optimal removal of the dysbiotic biofilm from the contaminated surface seems to be the primary objective to prevent or manage both peri-implant mucositis and peri-implantitis (15, 16).

The treatment approach usually depends on the severity of the defect:

Mucositis and slight peri-implantitis can be managed by means of nonsurgical therapies which consists in a professional mechanical debridement with or without the addition of antimicrobials. However, in moderate and advanced peri-implantitis, non-surgical therapies do not clinically show sufficient improvement (17, 18)*.* In such cases, surgical therapies are required after non-surgical debridement. It allows to make an access flap and, depending on the configuration of the lesion, to go for a reconstructive and/or resective procedure (19).

The use of air-abrasive device in conjunction with a surgical or non-surgical approach has been shown to be a safe and effective technique of decontamination in the treatment of peri-implant diseases (20, 21).

The PERIOFLOW® device is used with a single-use plastic nozzle to get access and to debride the implant surface with a water/glycine powder mix.

Furthermore, glycine powder is at low-risk than sodium bicarbonate as it does not cause tissue damage neither interfere with the biocompatibility of titanium towards osteoblasts (22, 23).

Additionally, it has been shown that the long-term survival of an implant has been correlated to the soft tissue seal around the implant collar (24).

Therefore, it seems interesting to link this concept with the results of different studies that have highlighted the positive effects of using platelet rich fibrin (PRF) derived from the patient's blood in the healing and regeneration of injured soft tissues (25, 26).

More recently, a liquid formulation of this platelet concentrates has been proposed by reducing the speed and the time of centrifugation: injectable PRF (i-PRF) (27, 28). This autologous bioactive agent is initially composed of fibrinogen and thrombin which gradually turns into fibrin and forms a blood clot after approximately 15 min. The three-dimensional fibrin network contains a lot of platelets and leukocytes, which secrete supra-physiological concentrations of growth factors including platelet-derived growth factor (PDGF), insulin-like growth factor-1 (IGF-1), vascular endothelial growth factor (VEGF), and transforming growth factor-beta 1 (TGF-β1) up to 10 days.

i-PRF increases the migration, proliferation and spreading of gingival fibroblasts as well as the expression of messenger RNA of regeneration-associated genes (PDGF, TGF-B and collagen 1) (27–29). Thus, it would improve wound healing and regeneration.

Another advantage of i-PRF is its antimicrobial action (30)*.* It is probably due to the high number of platelets and leukocytes but the exact mechanism yet to be established (31). Considering the relatively low success of periimplantitis, it would be beneficial to develop a personalized medicine approach that leverages the patient's defense system to achieve effective decontamination in the lacunae of rough surfaces where access is limited. This study aimed to assess the clinical and radiographic effectiveness of non-surgical treatment for mild peri-implant lesions using glycine air-polishing in combination with i-PRF.

## 2 Materials and methods

### 2.1 Study population

Patients with at least one implant showing evidence of and slight inflammatory peri-implant lesion were recruited during the periodontal consultation in the department of periodontology at the Saint-Luc university hospital. Patients were included if they met all the following inclusion criteria:

(1) Adult patients (>18 year)
(2) Bleeding and/or suppuration on probing (BoP and/or SoP)
(3) Absence of implant mobility
(4) Radiographic bone loss visible and up to 4 mm (between implant shoulder and the bone level)
(5) Absence of/or controlled periodontitis (DPSI 2)
(6) No antibiotics (local or systemic) or antiseptics taken in the three months preceding the start of the study

Patients were excluded if they presented any of the following criteria:

(1) Smoking habits
(2) Systemic disease or treatment requiring antiobioprophylaxis which could influence the therapy [unbalanced diabetes (HbAc1 > 7.0%), inflammatory diseases, bisphosphonates, immunosuppressors, certain cardiovascular conditions; radiotherapy]
(3) Antibiotic and/or antiseptic use in the previous three months
(4) Implant already treated
(5) Peri-implant bone loss > 4 mm (between implant shoulder and the bone level)

### 2.2 Study design

The present study was approved by the ethics committee of the Medical School of the Université catholique de Louvain, Brussels, Belgium in August 2020 (I-PRF_2020/11AOU/407). It was designed as a “*proof of concept”* prospective case series conducted over a 6-month follow up. The inclusion of patients was carried out from September 2020 till December 2021. Each included patient received detailed informations in advance about the study process, objectives and the duration of the follow-up and they had to sign an informed consent agreement. Also, for all patient an individual oral hygiene instruction (modified Bass technique, interdental brushes and floss) and a supragingival cleaning were given.

Recruitment, treatment, and follow-up were completed between September 2020 and May 2022.

#### 2.2.1 Periodontal records

All the following clinical parameters were recorded at baseline, 3 and 6 months after the treatment by the same examiner (LD) using a calibrated periodontal probe (0.2N, 20 g) (WHO DB765R, Aesculap, Tuttingen, Germany) at 6 points around the implant: Plaque Index (PI), Bleeding on Probing (BoP), Suppuration on Probing (SoP), Probing Pocket Depth (PPD), Relative Attachment Level (RAL) and gingival recession.

#### 2.2.2 Radiographic records

Bone level was measured with the Sidexis XG 2.52 program (Sirona Dental Systems GmbH, Germany) at baseline and at 6 months with intra-oral radiographs using the long cone paralleling technique, a phosphor plates (74321; Durr Dental AG, Bietigheim-Bissingen, Germany), a sensor holder (Eggen-holder/Super-Bite blocks; Kerr Dental, Orange, CA, USA) customized with a silicon bite (PERFEXIL PLATINIUM, Septodont, France). The distance between the bone level and the implant shoulder was measured in millimeters, mesially and distally, for each implant by the same investigator (LD). The delta between these two measurements is calculated.

#### 2.2.3 Calibration

One examiner (LD) carried out all clinical and radiographic measurements on patients who were not included in this study. The examiner was calibrated in measurements of PPD using a calibrated periodontal probe (0.2N, 20 g) (WHO DB765R, Aesculap, Tuttingen, Germany). A total of 60 sites out of the 66 measured for the calibration were within 1 mm of each other on the two occasions, resulting in an intraexaminer agreement of 90%. The radiographic bone levels measurements were made with the Sidexis XG 2.52 program (Sirona Dental Systems GmbH, Germany). Of the 16 sites measured by the examiner, all of them were within 0.5 mm of each other on the two occasions, resulting in an intraexaminer agreement of 100%.

### 2.3 Non-surgical procedure

For all patients a non-surgical decontamination of the implant surface was performed with the use of glycine air-polishing device under local anesthesia (Septanest Normal 1.8 ml 4% articaine, 1/200,000 adrenaline, Septodont, NV-SA).

**Figure 1:**
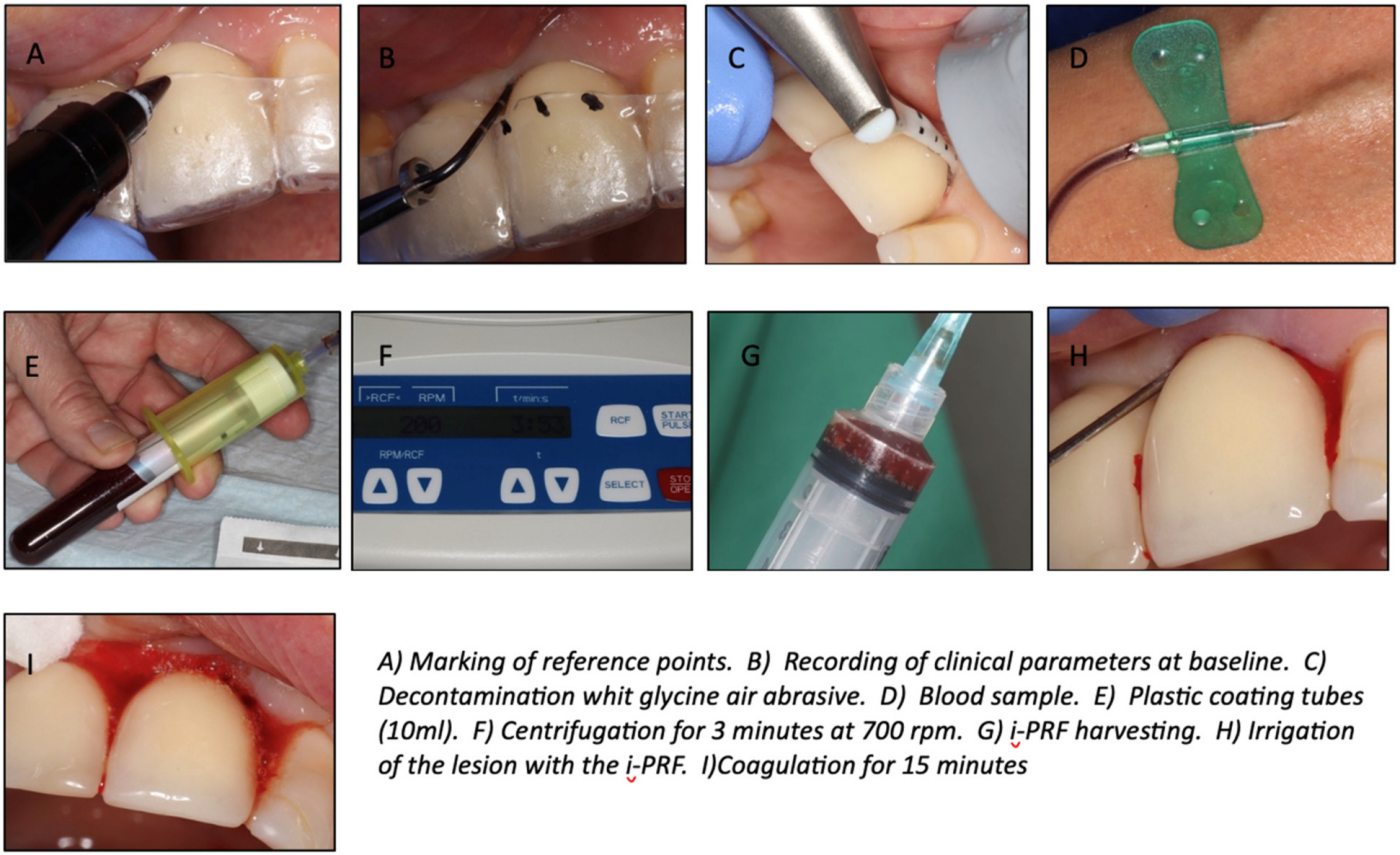
Clinical illustrations of the procedure.

A millimetric plastic nozzle (Perio-Flow nozzle; EMS, Nyon, Switzerland) (length 1.7 cm, Ø 0.8 mm at the tip) fixed on a handpiece (Perio-flow EL-354#, EMS) was inserted into the pocket tangential to the implant surface. The amino acid glycine powder (Air-Flow Perio powder, EMS) was projected at 4 sites (M—V—D—P/L) from the coronal to the apical part of the implant using a circular motion during 5 s on each site, as recommended by the manufacturer (Figure 1).

Afterwards, the pocket was rinsed with saline to remove particles and most of the biofilm (31)*.* Then, a sterile compress was placed all around the implant neck as to avoid recontamination.

With the consent of the patient, a whole blood sample was then taken by a nurse by using two plastic coated tubes (10 ml each). These tubes were immediately placed opposite each other for centrifugation into the IntraSpin™ system device (Intra-Lock Inc., FL, United States) at a speed of 700 rpm for 3 min (27).

After this procedure, the i-PRF liquid surnatant was collected directly by using a 10 cL sterile plastic syringe and applied afterwards in the submucosal peri-implant pocket. Then the patient was instructed to wait 15 min for the fibrin clot formation.

Post-operative care consisted in no brushing and cold diet for 24 h and then starting rinsing the next day with a mouthwash containing 0,2% chlorhexidine digluconate solution (Corsodyl, GlaxoSmithKline Consumer Healthcare, Buhl, Germany) 10 ml twice a day during one minute for ten days. Paracetamol (500 mg) was recommended if needed.

### 2.4 Statistical analysis

Clinical and radiologic parameters were collected throughout the study and results were expressed as means with standard deviations. The primary outcome was the PPD and was considered as statistically significant at alpha = 0.05.

Statistics were performed on the mean of each parameter. The evolution of the data between baseline and 3 months, 3 months and 6 months, and baseline and 6 months was assessed by a repeated measures ANOVA followed by a *post-hoc* Bonferroni test.

The calculations were performed using the JAMOVI version 2.3.3.

## 3 Results

### 3.1 Patient selection/demographics

Ten subjects (*n* = 15 implants) were recruited in this study (three men and seven women). One patient dropped out before the beginning of the study. Therefore, nine subjects (three men and six women) with a mean age of 58 [SE ± 2.5] (range from 45 to 71 years) were analyzed in this study. A total of fourteen implants were treated, eleven in the maxilla and only three in the mandible. A history of periodontitis was recorded in 22%.

### 3.2 Implant parameters

**Figure 2:**
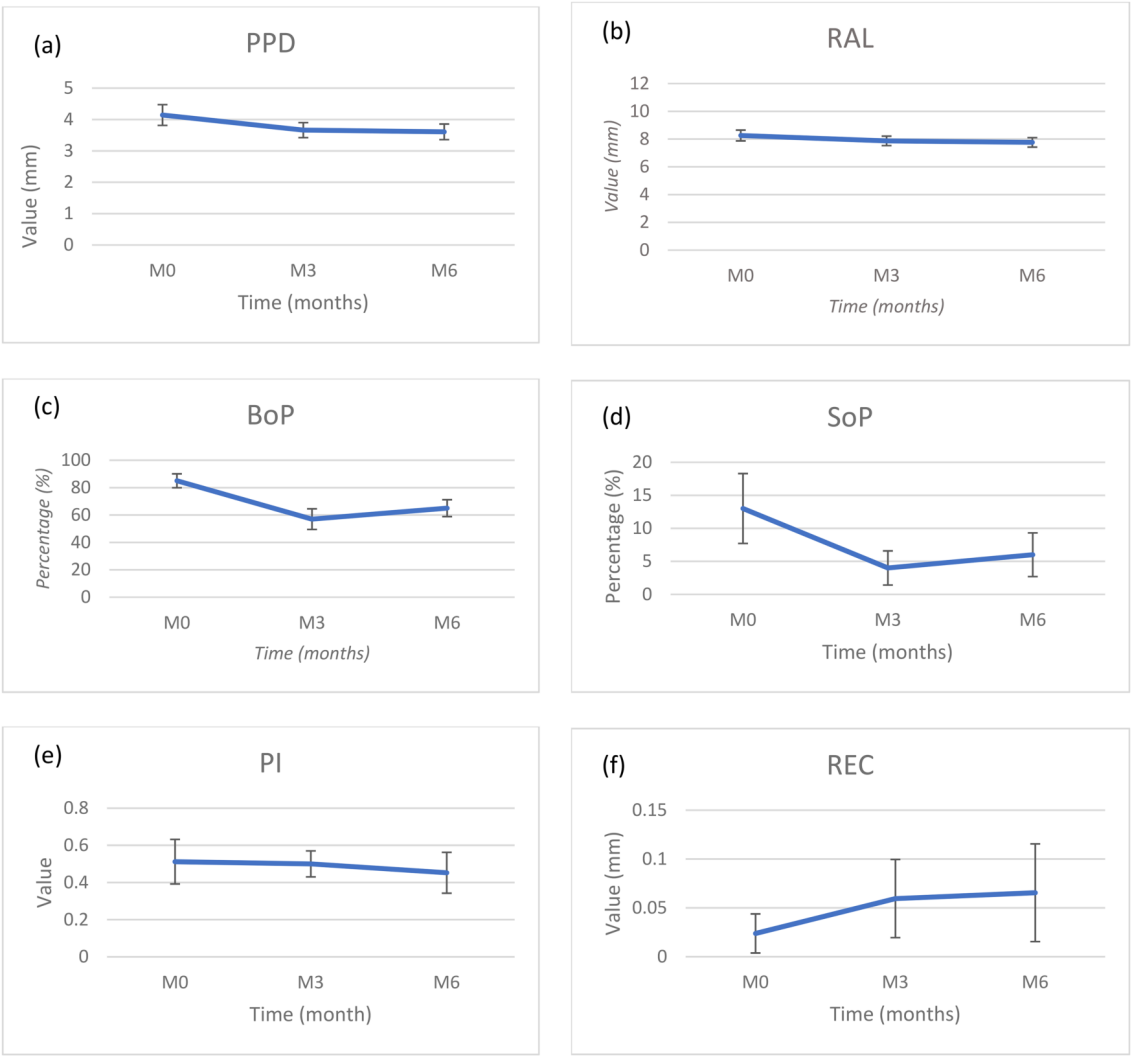
Changes of mean **(a)** probing pocket depth (PPD), **(b)** relative attachment level (RAL), **(c)** bleeding on probing (BoP), **(d)** suppuration on probing (SoP), **(e)** plaque Index (PI) and **(f)** recession (REC) at baseline, 3 months and 6.

The mean values of each parameter were collected at baseline, 3 months and 6 months (Table 1; Figure 2).

**Table 1:** Clinical and radiological data of implants at baseline, 3 months and 6 months follow-up.

Parameters	Baseline	3 months	6 months
PI, mean ± SE	0.51 ± 0.12	0.5 ± 0.07	0.45 ± 0.11*
BOP, % mean ± SE	84.5 ± 5.08	57.1 ± 7.56*	65.5 ± 6.17*
SOP, % mean ± SE	13.1 ± 5.29	3.57 ± 2.58	5.95 ± 3.31
PPD,mm mean ± SE	4.14 ± 0.33	3.65 ± 0.24	3.61 ± 0.25*
RAL,mm mean ± SE	8.26 ± 0.39	7.87 ± 0.34	7.76 ± 0.34*
REC, mm mean ± SE	0.02 ± 0.02	0.06 ± 0.04	0.06 ± 0.05
BL, mm mean ± SE	1.39 ± 0.37	/	1.12 ± 0.34*

* Significantly different from baseline (*p* < 0.05, ANOVA, Bonferroni).

Plaque index around implants decreased during follow-up to a mean value of <0.5.

Mean BoP values were significantly decreased from 84.5 ± 5.08% to 57.1 ± 7.56% (*p* < 0.05) after 3 months and then increased slightly but not significantly to 65.5 ± 6.17% (*p* < 0.05) at 6 months. Mean number of sites with SoP were not significant and followed the same pattern with a decrease from 13.1 ± 5.29% to 3.57 ± 2.58% in the first 3 months and then increased to 5.95 ± 3.31% at 6 months (*p* < 0.05).

Mean PPD reduction and RAL gain were not significant during the first 3 months (0.48 ± 0.24 mm and 0.38 ± 0.2 mm, respectively) but these values continued to improve until a significant difference between baseline and 6 months (0.53 ± 0.22 mm and 0.49 ± 0.18 mm, respectively). Mean PPD was 3.61 ± 0.25 mm (*p* < 0.05) at M6, and RAL gain was significantly improved at 6 months (7.76 ± 0.34 mm, *p* < 0.05). BL showed a significant improvement (1.12 ± 0.34 mm, *p* < 0.05). However, it is interesting to note that the major reduction in PPD and gain in RAL occurs during the first 3 months.

At 6 months, the mean mucosal recession was 0.06 ± 0.05 mm representing a nonsignificant increase of 0.04 mm from baseline. Most of the increase took place between baseline and 3 months.

Thus, concerning clinical parameters, only BoP, PPD and RAL improved significantly during the study period (*p* < 0.05).

**Figure 3:**
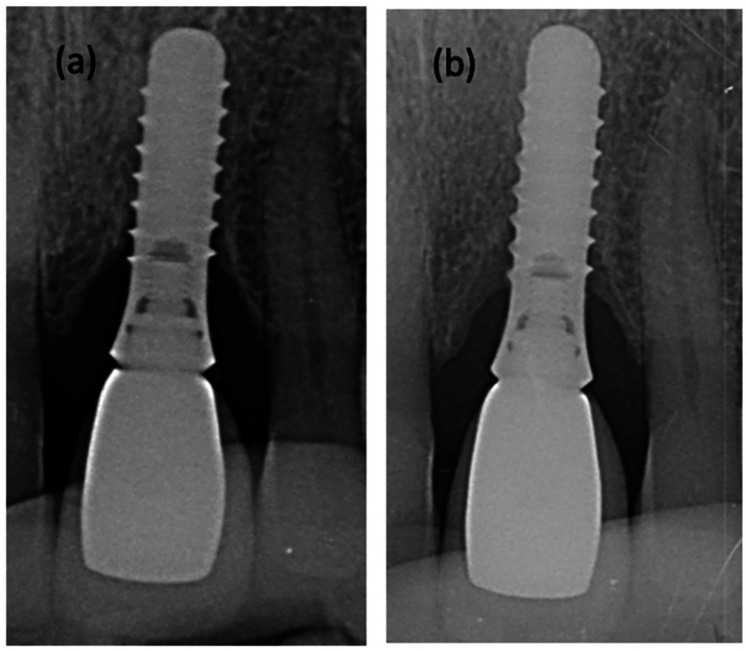
Radiographs of a peri-implant defect **(a)** before and **(b)** after non-surgical treatment with glycine air polishing and irrigation of i-PRf.

**Figure 4:**
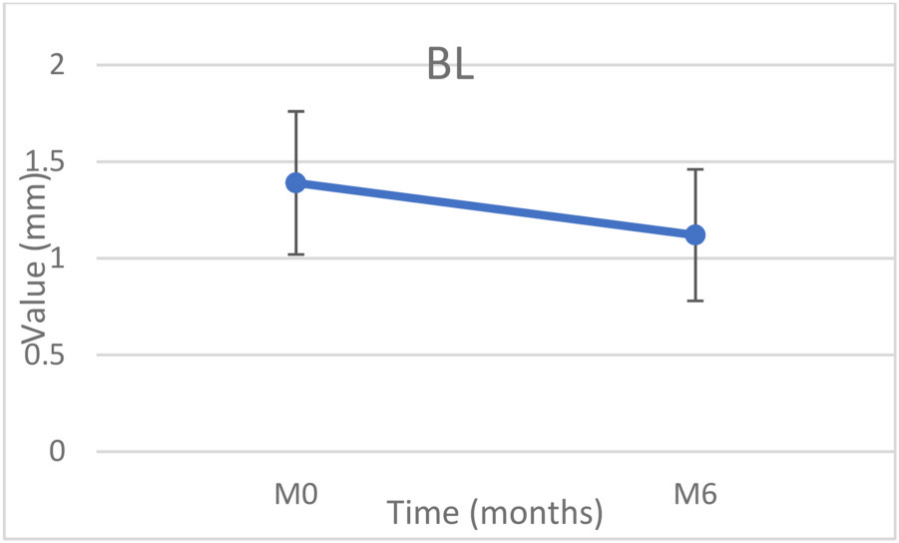
Changes of mean bone level (BL) at baseline and 6 months.

Bone levels around implants improved slightly but significantly between baseline and 6 months (+0.27 mm) (*p* < 0.05) (Figures 3, 4).

### 3.3 Treatment outcomes

Treatment outcomes were assessed using two different definitions. The first was proposed by Renvert et al. as a “positive treatment outcome” and was defined as a mean reduction in PPD ≥ 0.5 mm and no further bone loss between baseline and 6 months (20). The second is stricter as it includes BoP and SoP. It was proposed by Carcuac et al. as a “treatment success” and was defined as a PPD ≤ 5 mm, absence of bleeding or suppuration on probing at the implant site and no additional mean bone loss ≥ 0.5 mm between the baseline and six months follow-up (32).

According to the criteria of Renvert et al. 7 out of 14 implants were categorized as stable and the global positive outcome rate was hence 50%.

However, no implant was considered as successfully treated based on the Carcuac et al. criteria.

No patient reported any adverse effects following treatment.

## 4 Discussion

This prospective proof-of-concept case series aimed to evaluate the efficacy of non-surgical management of mild peri-implant inflammatory lesions using glycine air-polishing in combination with injectable platelet-rich fibrin (i-PRF).

As previously described, peri-implantitis is an inflammatory disease induced by the presence of a dysbiotic subgingival biofilm leading to peri-implant soft tissue inflammation and bone resorption. Therefore, the primary goal of the treatment is to remove biofilm to reduce the inflammation of the peri-implant tissues and thus stop the progression of bone loss and to maintain the implant function.

In this study, implant surface was non-surgically decontaminated using a glycine air-abrasive device followed by the irrigation of i-PRF in the peri-implant sulcus which aimed to stimulate the healing and regeneration of the damaged tissues.

Indeed, i-PRF has been described as a polymerized fibrin matrix containing a lot of platelets and leucocytes capable to release supra-physiological doses of growth factors (PDGF*,* IGF-1, VEGF, TGF-β1). Different mechanisms are involved in the healing process of peri-implant tissues (25):

• Platelets facilitates the fibrin clot formation, release cytokines/chemokines and growth factors capable of stimulating cell migration and proliferation within the fibrin matrix.
• Leucocytes are immune cells important in the host-defense response to the pathogen and are involved in tissue regeneration by secreting a large number of growth factors and cytokines associated with wound healing (e.g., IL-4, TNF-a, IL-6, IL-1) (29).
• TGF- β1 stimulates the proliferation of osteoblasts and promotes the synthesis of extracellular matrix components, including collagen type I and fibronectin, through both osteoblasts and fibroblasts. It is a key regulator of fibrosis and wound healing.
• PDGF regulates the migration, proliferation, differentiation and survival of mesenchymal cells. Therefore, it plays a role in physiological wound healing.
• VEGF is responsible for angiogenesis of tissues.
• IGF positively regulates the proliferation and differentiation of most mesenchymal cells. But also, it regulates the programmed cell death (apoptosis) by inducing survival signals protecting cells from apoptotic stimuli.

Currently, only one RCT has been performed evaluating the clinical outcomes of open flap debridement with or without PRF application in the treatment of peri-implantitis. This study showed that PRF increased PD reduction, clinical attachment gain and reduced mucosal recession after 3 and 6 months than open flap debridement alone (33)*.* Recently, an *in vitro* study showed that the application of L-PRF after a 0.9% NaCl rinsing significantly reduced the number of bacteria on the contaminated SLA titanium surface bringing a certain antimicrobial potential to PRF (34).

The results of the present study indicate that non-surgical treatment of slight peri-implantitis resulted in significant clinical and radiographical improvement as evidenced by reductions of bleeding on probing (BoP), probing pocket depth (PPD), and bone loss (BL), along with relative attachment level (RAL) gain at six months.

However, attention should be drawn to the fact the mean BoP significantly decreased between baseline and 3 months and then slightly but not significantly increased at 6 months, although it does not seem to have any impact on PPD and RAL. Also, PPD reduction and RAL gain at 6 months after therapy were in accordance with the results obtained in another study which uses an air-abrasive device in non-surgical treatment of initial to moderate peri-implantitis (35). The authors obtained a PPD reduction of 0.6 mm vs. 0.5 mm, a RAL gain of 0.4 mm vs. 0.5 mm and the mean BoP value followed the same trend.

When a composite outcome is assessed as to evaluate the effect of the proposed treatment option of slight peri-implantitis, 50% of treated implants were considered to have a positive outcome according to the criteria of Renvert et al. (20)*.* These results are in accordance with those of the above-mentioned study who showed a positive result of 47% following treatment of severe peri-implantitis with air-abrasive device.

None of the implants were considered as successfully treated according to the criteria set out by Carcuac et al. because all implants had at least one site with BoP at the 6-month evaluation visit (32).

Although studies evaluating the non-surgical treatment of peri-implantitis with a glycine air-abrasive device has shown encouraging results, the complete disease resolution was usually not achieved (36–38)*.* In cases with residual PPD ≥ 5 mm with BoP/SoP and radiographic bone loss of ≥2 mm after initial non-surgical treatment, surgical therapy should be considered (39). In this study, if we consider these criteria, 6 months after therapy only one implant out of 14 treated should undergo surgery. These results are not surprising as the included implants presented initially slight defects which seem to be easier to treat.

The others were placed in supportive therapy and advised about the importance of an effective plaque control to maintain the long-term outcomes.

As the results are comparable to those of Sahm et al., the application of i-PRF in the sulcus does not seem to influence the non-surgical treatment with a glycine air abrasion device (35).

A hypothesis that could explain our results is that the liquid aspect of i-PRF is difficult to stabilize. Indeed, it was applied directly after non-surgical debridement and bleeding could have contribute to remove it in part from the sulcus. As suggested by Kashefimehr et al., the i-PRF could have been applied 2 weeks after debridement to allow the peri-implant tissues to heal (40).

Another technique would have been to apply L-PRF membranes (more compact but containing less cells and growth factors) stabilized with an absorbable suture instead of i-PRF.

Apart from these considerations, it should be noted that a potential limitation of the present study is the absence of microbiological testing. In fact, a study reported on microbiological outcomes after a non-surgical treatment with an air-abrasive device showed that this procedure failed to reduce the bacterial load at 6 months (especially: *Aggregatibacter actinomycetemcomitans, Campylobacter gracilis, Campylobacter rectus, Eikenella corrodens, Leptothrichia buccalis, Staphylococcus anaerobius, Staphylococcus haemolyticus, Streptococcus gordonii, Streptococcus mutans, and Tannerella forsythia*) (41)*.* This could partly explain the residual BoP at this time point. Another unknown variable is the width of the peri-implant keratinized mucosa which was not evaluated and might potentially influence plaque accumulation and bleeding on probing (42, 43).

Furthermore, implant designs and surface characteristics were not considered in this study. This may have an impact on the biofilm formation and removal but also on the response of human gingival fibroblasts. It may be hypothesized that surface roughness may be involved in the healing process induced by i-PRF because of the different absorption capacity of proteins depending on the implant surface (29, 44).

This is a pilot study and further comparative studies could be needed to draw real conclusions.

### 4.1 Study limitations

The present study suffers from several limitations that need to be considered when interpreting the results. First, the study was designed as a case series and does not permit a comparison with another therapeutic approach. In addition, the limited sample size is another weakness of the present study. Furthermore, the periapical radiographs used only allow us to evaluate the mesial bone and distal bone loss but do not give any idea on the morphology of the defect.

## 5 Conclusion

In conclusion, the application of i-PRF after a subgingival debridement using a glycine air-polishing seems to be an interesting approach for the non-surgical treatment of slight peri-implantitis. Indeed, it allows significant improvement of clinical parameters such as BoP, SoP, PPD, RAL and a bone level stability for at least six months as shown in the present study. This allowed to avoid a surgical approach in most of the treated cases. On the other hand, if we consider that no bleeding on probing is needed to control the disease, none of the implants are considered successfully treated. However due to its interesting properties, further randomized clinical trials are needed to assess its additional clinical interest when compared to the air polishing alone.

## Data Availability

The raw data supporting the conclusions of this article will be made available by the authors, without undue reservation.
